# Isothermal Microcalorimetry, a New Tool to Monitor Drug Action against *Trypanosoma brucei* and *Plasmodium falciparum*

**DOI:** 10.1371/journal.pntd.0001668

**Published:** 2012-06-05

**Authors:** Tanja Wenzler, Andrea Steinhuber, Sergio Wittlin, Christian Scheurer, Reto Brun, Andrej Trampuz

**Affiliations:** 1 Medical Parasitology and Infection Biology, Swiss Tropical and Public Health Institute, Basel, Switzerland; 2 University of Basel, Basel, Switzerland; 3 Infectious Diseases Research Laboratory, Department of Biomedicine, University Hospital Basel, Basel, Switzerland; Institute of Tropical Medicine, Belgium

## Abstract

Isothermal microcalorimetry is an established tool to measure heat flow of physical, chemical or biological processes. The metabolism of viable cells produces heat, and if sufficient cells are present, their heat production can be assessed by this method. In this study, we investigated the heat flow of two medically important protozoans, *Trypanosoma brucei rhodesiense* and *Plasmodium falciparum*. Heat flow signals obtained for these pathogens allowed us to monitor parasite growth on a real-time basis as the signals correlated with the number of viable cells. To showcase the potential of microcalorimetry for measuring drug action on pathogenic organisms, we tested the method with three antitrypanosomal drugs, melarsoprol, suramin and pentamidine and three antiplasmodial drugs, chloroquine, artemether and dihydroartemisinin, each at two concentrations on the respective parasite. With the real time measurement, inhibition was observed immediately by a reduced heat flow compared to that in untreated control samples. The onset of drug action, the degree of inhibition and the time to death of the parasite culture could conveniently be monitored over several days. Microcalorimetry is a valuable element to be added to the toolbox for drug discovery for protozoal diseases such as human African trypanosomiasis and malaria. The method could probably be adapted to other protozoan parasites, especially those growing extracellularly.

## Author Summary

Microcalorimetry is a technology developed to record minute changes in temperature as a result of physical, chemical or biological reactions over time. The method has been applied to bacterial and eukaryotic cells and it was found that the metabolic activity of living cells in a culture medium produces enough heat flow to be measured. Protozoan parasites, some of which cause tropical diseases such as African sleeping sickness or malaria, are larger cells than bacteria and are metabolically very active. We explored the applicability of heat flow measurement to follow the growth of a parasite population and to study the effect of drugs. We first established optimal parameters for obtaining heat flow curves of a growing parasite culture. Then we added antiparasitic drugs at two concentrations and followed the heat flow curves over several days. Thus we could determine the time of onset of drug action and the time until all parasites stopped producing heat (time to kill). The microcalorimeter measurements once per second allowed a continuous monitoring of changes in the parasite population. This novel tool is accurate and simple to use, and will certainly prove to be of great value for the discovery and development of new drugs for protozoan parasites.

## Introduction

Human African trypanosomiasis (HAT), also known as African sleeping sickness, and malaria are important tropical diseases caused by protozoan parasites. HAT threatens millions of people living in sub-Saharan Africa [1]. In recent years, the number of cases dropped due to improved control measures such as trapping of tsetse flies, active surveillance and appropriate treatment of patients, and is currently estimated at 30,000 cases annually [2]. However, the disease may reemerge, if control is neglected. African sleeping sickness is fatal without treatment, so the availability of effective drugs is vital. Malaria has a higher public health impact with 225 million infections and almost 800'000 deaths annually [3]. The most affected populations are children and pregnant women in Africa. Effective drugs are available for prophylaxis and treatment, but drug resistant parasites represent a major challenge. Therefore, new drugs for both diseases are needed on a continuous basis particularly since no effective vaccine is yet available for either of these diseases, so drug development is of crucial importance.

Drug discovery and development requires rapid methods for screening large number of compounds. For both trypanosomes and malaria parasites, in vitro drug activity tests are available. These are routinely performed in 96-well microtiter plates with a drug exposure time of 72 hours. For *Trypanosoma brucei* spp. bloodstream forms are cultivated axenically. Parasite inhibition is determined in a simple and cost-effective way using the viability marker Alamar blue (resazurin) [4]. *P. falciparum* is cultured as asexual erythrocytic stages, and parasite growth inhibition is classically assessed by measuring the uptake of tritium-labelled hypoxanthine [5]. Using these assays, the antiprotozoal activity of added compounds, expressed as 50% inhibitory concentration (IC_50_) can be determined. These methods can also be used to determine the time of onset of drug action and the time to kill, which are of great importance for subsequent in vivo studies. However, following changes over time using these currently available in vitro tests is not very accurate and is particularly labor intensive.

An alternative method of estimating growth inhibition is isothermal microcalorimetry. This nonspecific technique allows direct measurement of heat generated by biological processes in living cells. Growth of microorganisms results in an increase of heat flow over time which is documented by a continuous real-time electronic signal. The method has already been used to study heat production of bacteria, mammalian cells and worms [6]–[10]. For example, bacteria produce on average 1–3 pW heat per viable cell [6], [11]. The detection time depends on the sensitivity of the instrument as well as the initial number of living cells, their growth rate and the amount of heat produced per cell [12], [7]. To our knowledge, this technique has not been applied yet to any pathogenic protozoa.

In the present study, we established microcalorimetry as a new tool for a rapid determination of effects of drugs on *Trypanosoma brucei rhodesiense* and *Plasmodium falciparum*. We used the real-time measurements of metabolic heat flow produced by these protozoan parasites, to measure the time of the onset of action at different drug concentrations and also the time to death of the parasite population.

## Materials and Methods

### Culture of *T. b. rhodesiense* and preparation of calorimetry ampoules

Bloodstream forms of the *T. b. rhodesiense* strain STIB900 were cultivated in Minimum Essential Medium with Earle's salts, supplemented according to Baltz et al. [13] with the following modifications: 0.2 mM 2-mercaptoethanol, 1 mM sodium pyruvate, 0.5 mM hypoxanthine and 15% heat-inactivated horse serum. For calorimetry, trypanosomes were washed and diluted with fresh culture medium to give the desired initial cell density then transferred to 4 ml sterile glass ampoules which were hermetically sealed with a rubber septum.

For the determination of a suitable cell density to use to obtain growth curves, ampoules were filled with 3 ml trypanosome culture containing 10^4^, 10^5^ and 10^6^ cells/ml initial densities, each in triplicate. Culture medium without trypanosomes served as negative control. Continuous heat measurements (1/sec) were conducted over a period of up to 6 days. For determination of parasite densities at different time points, small aliquots (≈50 µl) were collected through the rubber septum of the hermetically closed ampoules using a 1 ml syringe. Cell counting of motile trypanosomes was performed microscopically using a Neubauer chamber.

The influence of the sample volume was evaluated using an initial density of 10^5^ cells/ml, and 1 ml, 2 ml and 3 ml of culture medium each in triplicate.

The standard drugs suramin, pentamidine and melarsoprol were selected to monitor drug action. Eflornithine was excluded because of its weak in vitro activity against African trypanosomes. We used a multiple of the IC_50_ value of each drug since time to kill can not be determined with an IC_50_ (determined over 72 hrs) or lower concentrations. For the investigation of drug activity, trypanosome cultures were diluted with fresh culture medium to a density of 10^5^ cells/ml. Each ampoule was filled with 3 ml cell suspension and supplemented with suramin, pentamidine or melarsoprol at concentrations corresponding to 5× IC_50_ or 25× IC_50_ (for actual concentrations in ng/ml see table 1). Each measurement was performed in triplicate. The IC_50_ values were determined prior the experiment as previously described [14].

**Table 1:** Heat flow parameters of *T. b. rhodesiense* culture exposed to pentamidine, melarsoprol or suramin at two concentrations.

Drugs	Concentration	Onset of action (hours)	Time to peak (hours)	Peak heat flow (µW)	Time to base level (hours)
Drug-free control	-	-	30	8.0	120
Pentamidine	5× IC_50_ (8.5 ng/ml)	≤3	3	2.2	28
Pentamidine	25× IC_50_ (42.5 ng/ml)	≤3	3	2.0	22
Melarsoprol	5× IC_50_ (11.0 ng/ml)	6	9	4.1	>120
Melarsoprol	25× IC_50_ (55.0 ng/ml)	≤3	3	1.7	12
Suramin	5× IC_50_ (833 ng/ml)	5	9	4.3	42
Suramin	25× IC_50_ (4165 ng/ml)	5	9	4.0	32

### Culture of *P. falciparum* and preparation of calorimetry ampoules

The *P. falciparum* strain NF54 was cultivated as previously described [15], [16]. An aliquot of 0.5 ml of unsynchronized *P. falciparum* culture with 10% parasitemia and 5% hematocrit was mixed with fresh human erythrocytes and culture medium to give the desired initial parasitemia and 5% hematocrit.

Samples with an initial parasitemia of 1.0, 0.5, 0.25 and 0.125% were tested to find the optimal initial parasitemia for the evaluation of fast-acting drugs. In addition, the influence of the volume on the thermal profile was evaluated using 4 ml-ampoules filled with 0.5, 1.0, 2.0 or 3.0 ml of unsynchronized culture with an initial parasitemia of 0.5% and 5% hematocrit. This was done because erythrocytes settle rapidly, so different volumes of culture medium in a 4 ml ampoule might influence parasite development owing to differences in oxygen supply and availability of nutrients. Ampoules filled with non-infected erythrocytes (5% hematocrit) were used in triplicate as negative controls. Continuous heat measurements were conducted over a period of 5 days. Parasitemia was assessed by microscopic counting of Giemsa-stained smears prepared from samples aspirated at defined time points with a syringe through the rubber septum of the closed ampoules.

For the drug test, aliquots of stock solutions of the drugs were mixed with fresh *P. falciparum* culture to give the desired concentration and then distributed into sterile calorimetry ampoules. The antiplasmodial drugs chloroquine, artemether and dihydroartemisinin were tested at concentrations corresponding to 3× and 10× the published IC_50_ values [17]. Dilutions of the 10 mg/ml stock solutions were freshly prepared in culture medium, immediately before the start of the experiment.

### Calorimetric equipment and measurements

An isothermal calorimetry instrument (Thermal Activity Monitor, Model 3102 TAM III, TA Instruments, New Castle, DE, USA) equipped with 48 channels was used to measure heat flow continuously at 37°C. The temperature of the instrument was maintained within 0.00001°C. The calorimetric sensitivity according to the manufacturer is ±0.2 µW. The calorimeter continuously measured heat generated or absorbed by test or control samples in air-tight 4 ml glass ampoules sealed with a rubber septum. The gas phase was ambient air. For each series of measurements, ampoules were introduced into the calorimeter and remained at least 15 minutes in the thermal equilibration position at 37°C before they were lowered into the measurement position. Due to short-term thermal disturbance after introduction of the samples into the measuring position of the calorimeter the heat signal of the first 1 hour was considered unspecific. For the study of the relationship between heat flow events and the number of parasites in the sample, ampoules were removed at defined time points and aliquots of the samples were aspirated with a 1 ml syringe through the rubber septum, and the parasites counted.

### Analysis of calorimetric data

Thermal changes in each ampoule were recorded as a continuous electronic signal (in Watts), which is proportional to the heat production rate. After measurement, data were reduced from 1 second to 1 minute intervals and exported. Data reduction is optional and can be set individually to any degree after each experiment. Data analysis was accomplished using the manufacturer's software (TAM Assistant, TA Instruments, New Castle, DE, USA) and Origin 7.5 (Microcal, Northampton, MA, USA). For the analysis of time points one hour of incubation time was added to the time of the calorimetric measurement. This time was needed for preparation of the ampoules, their transfer from the bench to the calorimeter and for equilibration in the calorimeter prior the measurement.

## Results

### Influence of initial density of *T. b. rhodesiense* cultures on heat flow

We plotted heat flow (in µW) over time in all present experiments as the heat flow data can be used as a proxy for the number of viable trypanosome cells.

**Figure 1:**
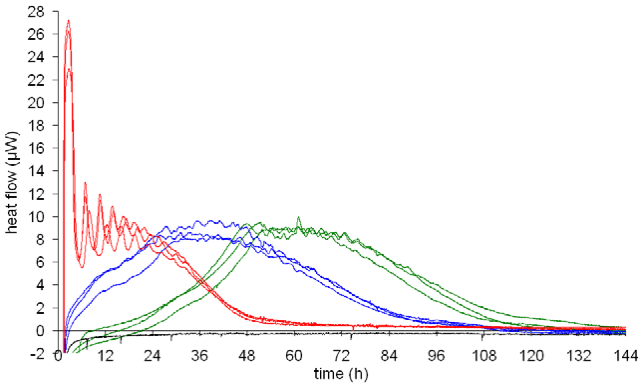
Calorimetric measurements of *T. b. rhodesiense* at different initial densities. Heat flow curves of *T. b. rhodesiense* at initial densities of 10^6^ cells/ml (red), 10^5^ cells/ml (blue) and 10^4^ cells/ml (green) in medium without the addition of drugs. All curves are means of triplicate measurements. The curves with the same initial trypanosome densities were measured in three independent experiments performed on different days.

Figure 1 shows the heat flow over time from 3 ml samples in closed 4 ml glass ampoules of *T. b. rhodesiense* containing three different initial trypanosome densities of 10^4^, 10^5^ and 10^6^ cells/ml. With an initial trypanosome density of 10^4^ cells/ml, a lag phase was observed before the exponential growth phase started. A stationary phase was reached after 48 hours. After 72 hours, the culture overgrew, which led to a decline of the heat flow. With an initial trypanosome density of 10^5^ cells/ml, the exponential phase started immediately, and the stationary phase as well as the dying off phase was observed one day earlier than with 10^4^ cells/ml. The maximum trypanosome density in the ampoules containing the cells was reached at maximum heat flow of around 8 µW.

At the lower initial trypanosome densities of 10^4^/ml and 10^5^/ml, the heat flow increased continuously and only minor amplitude oscillations occurred when the maximum heat flow was reached (above 8 µW). With 10^6^ cells/ml initial trypanosome density, unexpected heat flow oscillations were noted during the first 24 hours. A first peak of up to 26 µW was observed 2.5 hours after the start of the experiment (Figure 1). Then, the heat flow dropped and an oscillating heat flow started with continuously decreasing amplitudes during the first 24 hours. After 48 hours, the culture overgrew and the heat flow was close to the base level.

Parallel determinations of cell counts showed that the increase in heat production was consistent with the increase in cell numbers and the heat flow curves were similar to the growth curves obtained by cell counting (data not shown). The following decrease in heat production is due to decreasing numbers of viable cells combined with decreasing metabolic activity of the cells over time. The intra- and inter-experimental reproducibility was evaluated by running three independent measurements. Each was performed on different days, in triplicates and with trypanosome cultures freshly diluted to the desired densities. As expected, the reproducibility was higher within one experiment than between experiments. However, reproducibility was good also between different experiments, as illustrated in Figure 1, which shows the mean heat flow curves of triplicates of each experiment.

In the subsequent experiments for monitoring drug action, a trypanosome density of 10^5^ cells/ml was chosen to avoid the lag phase observed with 10^4^ cells/ml and to avoid the strong oscillations originating from samples with 10^6^ cells/ml initial density. A further disadvantage of using 10^6^ cells/ml is that only a very limited growth is possible, since the maximum trypanosome density under ideal culture conditions is ∼2×10^6^ cells/ml.

The time-courses for heat flow using 0.5 ml, 1 ml, 2 ml and 3 ml of cell culture at an initial density of 10^5^ cells/ml were all in a similar range (data not shown). As the largest volume contained the highest total number of cells and therefore produced the highest heat flow peak, a volume of 3 ml was chosen for the following experiments.

### Influence of antitrypanosomal drugs on the heat flow of *T. b. rhodesiense*

The standard drugs suramin, pentamidine and melarsoprol were selected to monitor drug action.

**Figure 2:**
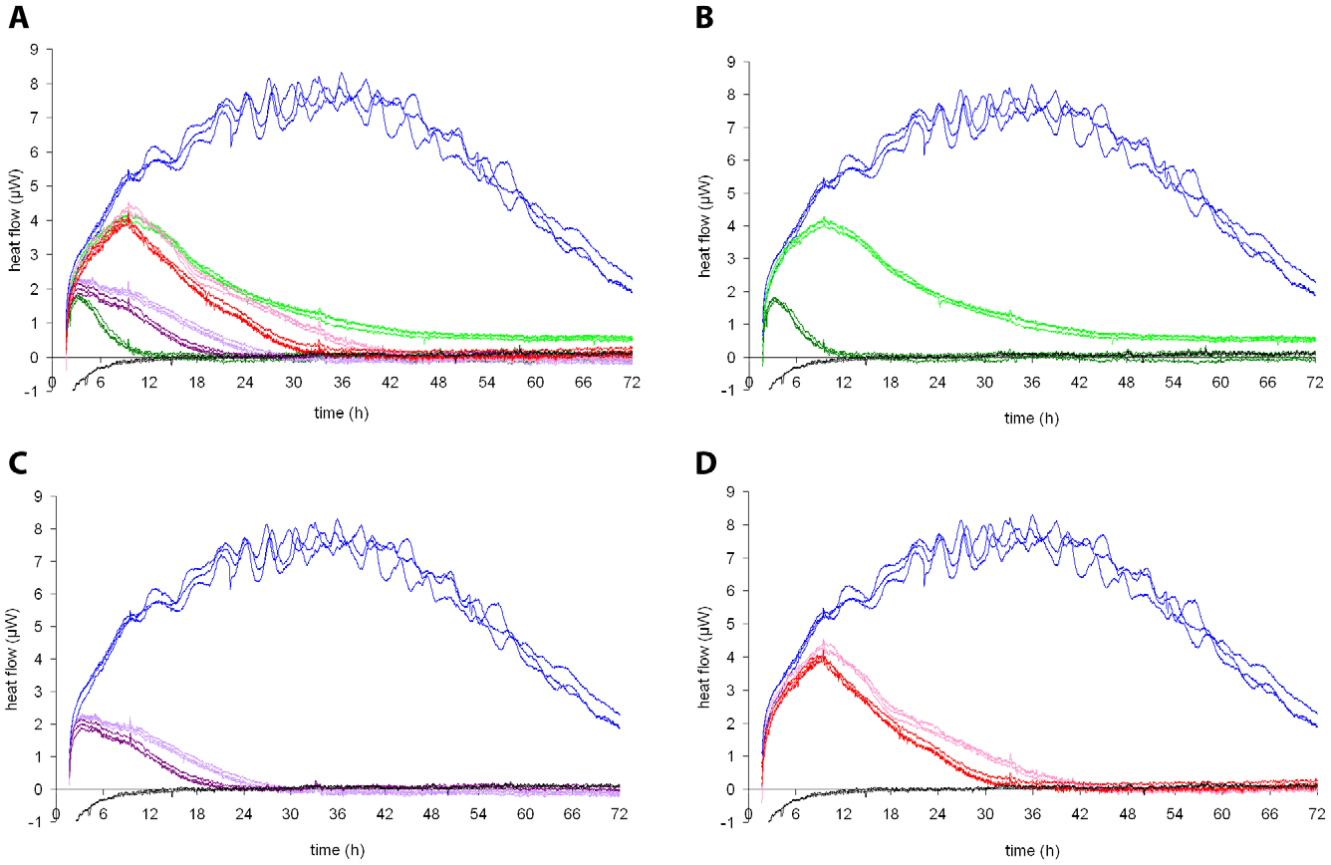
Calorimetric measurements of *T. b. rhodesiense* exposed to different drugs. Heat flow curves of *T. b. rhodesiense* at initial densities of 10^5^ cells/ml in medium without drugs (blue) or with drugs at two different concentrations, and a medium control without trypanosomes (black). All measurements were performed in triplicate. A: summary, all three compounds at 5× IC_50_ and 25× IC_50_; B: Melarsoprol at 5× IC_50_ (light green) and 25× IC_50_ (dark green); C: Pentamidine at 5× IC_50_ (light violet) and 25× IC_50_ (dark violet); D: Suramin at 5× IC_50_ (magenta) and 25× IC_50_ (red).

The heat flow of cultures containing melarsoprol (Figure 2B), pentamidine (Figure 2C) and suramin (Figure 2D) in concentrations corresponding to 5× IC_50_ and 25× IC_50_ was measured in parallel to that of control cultures containing no drug. Whereas the heat signal of the control cultures increased continuously to a peak value of 8 µW after 24–36 hours, curves for all drug containing samples reached markedly lower peaks, at earlier time points (Figure 2A and Table 1). Inhibition depended on the drug used, and its concentration. During the first 3 hours of measurement, the heat production of all samples increased in a similar way. The onset of action of the drugs was marked by a divergence in the continuously increasing heat flow curves of the drug containing specimens from the curves for the control specimens (blue curves). For all three drugs, the onset of action was within the first 6 hours of drug incubation (Figure 2A and Table 1). Then after the peak the heat flow continuously declined over a few hours until the heat production was reduced to the level of the sterile medium control (base level). The time required to reach this point, when the parasite culture was completely inactivated, was the time to kill. The fastest antitrypanosomal effect was observed with melarsoprol at 25× IC_50_, with a decline in heat production starting within the first 3 hours and reaching base line after 12 hours of incubation. The slowest acting drug among those tested was suramin. The heat production of suramin-treated trypanosomes started to decline after 9 hours of drug incubation. The heat flow was reduced to the base level after 32 hours at 25× IC_50_ and after 42 hours at 5× IC_50_ concentrations.

### Influence of different culture volumes of *P. falciparum* on heat flow

Blood stages of *Plasmodium falciparum* were cultured using human erythrocytes in culture medium [15], [16]. In our samples, erythrocytes settled and accumulated at the bottom of the vial within a few hours. This could tend to reduce the availability of both oxygen and nutrients, and produce an accumulation of metabolites. Both these effects might lead to decreased viability of the cells. The cultures used to evaluate the influence of different sample volumes in the 4 ml closed glass ampoules all had the same concentration of cells and the same initial parasitemia. The largest volume tested contained the highest overall number of cells and thus produced the highest amount of heat. The course of the heat flow curves was more or less similar for the different sample volumes tested (data not shown). However, with 3 ml or 2 ml samples, there was an initial pre-peak at 3–4 hours followed by a sharp drop at 7 hours before the heat flow increased again to reach the main peak, whereas with the 1 ml samples the heat flow signal increased steadily from the start of measurement until the main peak at ∼10 µW, and the heat flow curves of samples containing infected erythrocytes could easily be distinguished from those for uninfected erythrocytes (controls) immediately after the start of the measurement. This was not the case for the samples with 0.5 ml, the lowest volume tested. We therefore chose a volume of 1 ml at 5% hematocrit for the following experiments.

### Influence of initial density of *P. falciparum* on heat flow

**Figure 3:**
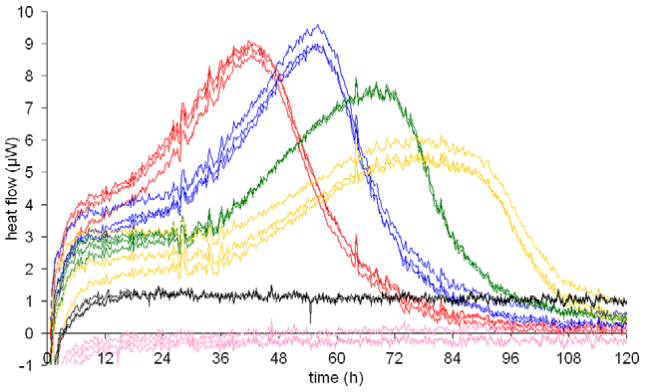
Calorimetric measurements of *P. falciparum* at different initial parasitemia. Heat flow curves of *P. falciparum* in 1 ml culture samples with 5% hematocrit and initial parasitemia of 1% (red), 0.5% (blue), 0.25% (green) or 0.125% (yellow). Control measurements were performed with samples containing uninfected erythrocytes only (black) and culture medium without any cells (pink). All measurements were performed in triplicate.

The heat production of 1 ml specimens containing erythrocytes (5% hematocrit) infected with *P. falciparum* at initial levels of parasitemia varying between 0.125%–1.0% increased, reached a peak and declined afterwards at all densities. The average time to peak was dependent on the initial parasitemia with the shortest time to peak being measured in the specimens with the highest initial parasitemia (1.0%: 43 h, 0.5%: 57 h, 0.25%: 72 h, 0.125%: 81 h) (Figure 3). Microscopic observation of the culture by Giemsa staining confirmed that the decreasing heat flow after the peak was due to dying of the parasites (data not shown). For the subsequent experiments we chose an initial parasitemia of 0.5%. With this concentration the heat flow reached a maximum peak value (9 µW) among the initial parasitemia levels tested. The time to reach the peak was approximately 57 hours.

### Influence of antimalarial drugs on the heat flow of *P. falciparum*

**Figure 4:**
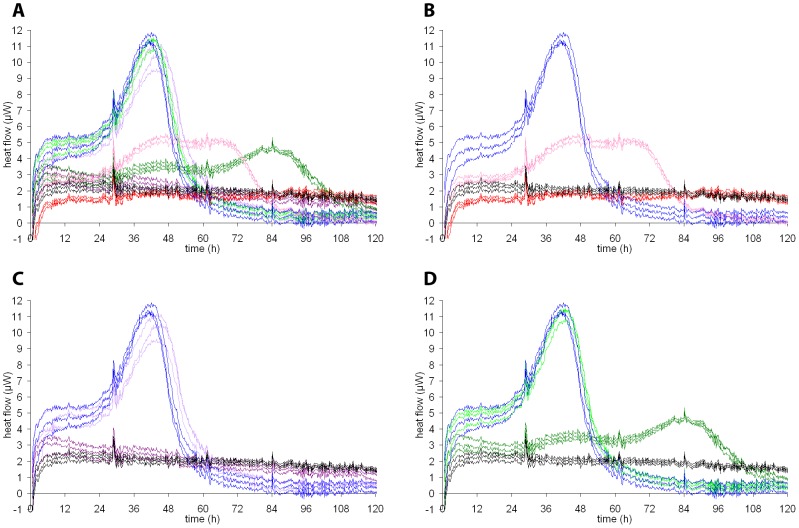
Calorimetric measurements of *P. falciparum* exposed to different drugs. Heat flow curves of *P. falciparum* in culture with 5% hematocrit and an initial parasitemia of 0.5% without drugs (blue) or with drugs at two different concentrations. Control measurements were performed with samples containing uninfected erythrocytes only (black). All measurements were performed in triplicate. A: summary, all three compounds at 3× IC_50_ and 10× IC_50_; B: Dihydroartemisinin at 3× IC_50_ (magenta) and 10× IC_50_ (red). C: Chloroquine at 3× IC_50_ (light violet) and 10× IC_50_ (dark violet); D: Artemether at 3× IC_50_ (light green) and 10× IC_50_ (dark green).

The influence of three standard drugs was measured using 1 ml of a non-synchronous *P. falciparum* culture with 0.5% initial parasitemia. The previously determined IC_50_ values of 5.1 (±0.8) ng/ml for chloroquine, 1.2 (±0.1) ng/ml for artemether [17] and 0.76 (±0.04) ng/ml for dihydroartemisinin were taken as a reference. Figure 4 shows the heat flow curves of a typical measurement in triplicate containing drugs at 3× and 10× the IC_50_. At 10× IC_50_ all drugs reduced the heat flow compared to the increasing heat flow observed in the drug free control (Figure 4A). The curves were highly reproducible. The most effective drug tested was dihydroartemisinin at 10× IC_50_ (Figure 4B), where the heat flow curves blended with those of the negative controls, containing uninfected erythrocytes. Chloroquine at 10× IC_50_ started to repress the parasite-specific heat production around 6 hours after measurement started. Afterwards the heat signal decreased continuously and matched the signal from uninfected erythrocytes from 48 hours onwards (Figure 4C). At 10× IC_50_, artemether appeared to be the least effective antiplasmodial drug of the ones tested. During 30 hours of measurement the heat flow signal was comparable with that of the samples with chloroquine at 10× IC_50_. However, plasmodial activity could be observed afterwards by an increasing heat flow leading to a low peak of 4.5 µW at 85 hours (Figure 4D). At the lower concentrations (3× IC_50_), dihydroartemisinin was again more effective than chloroquine and artemether, which both led to similar heat flow curves comparable to those for the drug-free control.

## Discussion

The calorimetric approach described here, offers the possibility of obtaining real-time measurements in up to 48 samples in parallel. This is not possible with conventional drug activity assays, where inhibition is measured at a single time point. Estimation of the time of drug action requires several assays with different drug exposure times, making such analysis highly labour-intensive. Isothermal microcalorimetry allows a more accurate determination of the onset of action and the time to kill based on the continuous measurement of the heat flow (1×/sec). Calorimetry is an unspecific tool, which cannot discriminate between metabolically inactive cells and dead cells, but it does allow the continuous monitoring of metabolic changes in a parasite population with little effort. It can be used to gain additional information when combined with established drug sensitivity assays.

In our study, we have plotted heat flow over time as the heat flow is proportional to microbial activity [6]. We considered that the heat flow correlates also with the number of viable (i.e., metabolically active) cells because metabolic activity of each trypanosome cell is expected to be more or less constant until it reaches the dying off phase with the decrease of metabolic activity per cell. A similar effect was observed with CHO 320 cells by Kemp et al [10]. An integration of the heat flow leads to a heat over time curve. Heat is proportional to total biomass produced or the quantity of a metabolic product released [6]. As we were interested in viable cells only, we used heat flow data (and not heat data) for our analysis.

In our microcalorimetric experiments, we found that the time to maximum heat flow varied according to the initial parasite density and the sample volume used. Optimization of these parameters led us to use a volume of 3 ml for *T. b. rhodesiense* with an initial trypanosome density of 10^5^ cells/ml and of 1 ml for *P. falciparum* with an initial hematocrit of 5% and a parasitemia of 0.5%. At these conditions, the heat flow signals were in the µW range which is well above the detection limit, which was specified by the manufacturer as 200 nW.

A limitation of this new methodology is the airtight sealing of the ampoules which is required for a proper measurement but could potentially affect the action of drugs, since no exchange of the gas phase or the culture medium is possible. Metabolic production of CO_2_ can lead to an acidification of the culture medium, as oxygen supply is limited. After a prolonged time of growth the conditions are likely to become non-physiological, therefore the period of accurate measurement is limited. After this, thermal effects might be misinterpreted. The samples were filled into the ampoules at normal laboratory conditions. Therefore the gas phase in each ampoule was normal ambient air rather than the special gas mixtures used for parasite cultures. An improvement of the culture conditions in the ampoules could be achieved by replacing the air in the ampoules by the gas used for trypanosome culture (5% CO_2_ in ambient air) or the special mixture used for malaria parasite cultures (4% CO_2_, 3% O_2_, 93% N_2_). However, no considerable deleterious effect of ambient air could be observed in our experiments over the maximum measurement period of 5 days. If further optimization of gas exchange is necessary for other experiments, a modified cap for the ampoules with a porous silicon rubber membrane could be used to allow gas exchange but no water evaporation [18].

The parasites used in our study are cultured under different conditions. African trypanosomes, as extracellular parasites, offer the advantage of axenic cultivation. There was therefore no background due to other cells to interfere with the heat flow signal, which allowed direct interpretation. *P. falciparum*, on the other hand, is an intracellular parasite. Non-infected erythrocytes produced a background heat level. They gave a rather constant heat flow between 1 and 2 µW. However, this background did not interfere with the interpretation of the heat flow curves produced by the parasite-infected erythrocytes (Figure 3). Another difference between the two parasites is the multiplication rate. Trypanosomes are mobile and replicate by binary fission, while *P. falciparum* after infecting the erythrocyte undergoes multiple replications and destroys the host cell. Erythrocytes tended to settle rapidly at the bottom of the ampoule, which may explain why smaller volumes (1 ml) of *P. falciparum* cultures in the calorimetric ampoules gave better signals.

When dense cultures of *T. b. rhodesiense* (10^6^ cells/ml) were used, they exhibited synchronized oscillations in heat production with a period of about 4 hours (Figure 1). These oscillations were highly reproducible even though the cultures themselves had not been synchronized prior to the experiments. Oscillations were also induced in samples with low initial trypanosome density when aliquots were taken for parasite counting or when the cell suspensions were mixed once they had reached a high trypanosome density. The oscillations were not dependent on the cell cycle as the generation time of *T. b. rhodesiense* is 8 to 9 hours. The oscillations may reflect underlying metabolic changes; glycolytic oscillations have been observed before in microcalorimetric studies by Lamprecht [19] in systems far from equilibrium. The author detected correlations of heat flow oscillations with NAD/NADH+ absorption in *S. cerevisiae* [19]. Analysis of the cause of heat flow oscillations in trypanosome cultures would be of interest but is beyond the scope of the current study. To avoid interference with drug inhibition by these oscillations, lower trypanosome densities were used for all experiments studying drug action.

Using the optimal conditions described, three antitrypanosomal and three antiplasmodial drugs were used to demonstrate that microcalorimetry is a helpful tool to monitor drug action against the two pathogenic protozoans on a real time basis. With only two concentrations per drug, we could observe the rate of action of each of the drugs tested and also the differences between them. Among the antitrypanosomal drugs, melarsoprol was found to be the fastest acting, followed by pentamidine and then by suramin. This ranking is in agreement with data obtained by the standard drug assays with different drug exposure times where the difference of IC_50_ values at 24 and 48 hours was smallest for melarsoprol (unpublished data). Similar studies have been performed with the antiplasmodial drugs. For *P. falciparum*, the microcalorimetric results are in agreement with data employing the standard [3H]hypoxanthine incorporation assay [20]. In our studies chloroquine and artemether showed pronounced parasite growth inhibition (≥94%) after incubations of 6 hours or longer at concentrations of 10× the IC_50_.

The method can be used with more than two different drug concentrations, which would enable the inhibition kinetics of a compound to be fully described, with an accuracy far greater than that of standard drug assays.

Another method which allows the determination of the time for a drug to exert its activity is real-time high content imaging. With this method, a higher throughput and more information can be obtained than with microcalorimetry. However, this results in a huge amount of data as pictures are taken instead of measuring a single value at each time point. Transmission light microscopy might not be sufficient to measure viability of trypanosomes, which are small and highly motile, or of plasmodia, which are intracellular, without any markers. Isothermal microcalorimetry has the further advantage that it is a label-free technique, therefore no interventions with the samples such as fixation, staining, or insertion of reporter genes in the parasites are required. Heat flow measurements can give information beyond viability and density such as indications of metabolic state [6], [19].

In spite of its many advantages, it is not likely that microcalorimetry will replace the routine screening assays which are currently used to determine antiprotozoal activities of new compounds. With conventional standard assays a higher throughput can be generated, using 96 well or even 384 well formats. However, microcalorimetry is a promising tool for gathering information about selected compounds beyond the IC_50_, such as the time until onset of action. Measuring this can be a challenge for fast acting compounds that begin to affect growth within the first 3 to 4 hours. In our studies with trypanosomes we found that the time for the preparation of the specimens, their transfer, and the equilibration time in the calorimeter could overlap with the time of onset of action. This problem could be reduced by using an injection system which allows equilibration in the calorimeter prior to the injection of the compounds (personal communication Matthias Rottmann).

The new approach to studying the effect of drugs, as described for the two model organisms *T. b. rhodesiense* and *P. falciparum*, may be applicable also for other protozoan parasites. Resistance or sensitivity analysis of different parasite isolates or screening for resistance development may be an additional interesting field for the use of microcalorimetry. Real-time drug inhibition data can be used in combination with pharmacokinetic and pharmacodynamic data as a helpful tool to predict the outcome of in vivo experiments in the field of drug discovery.
